# Triple-Vessel Spontaneous Coronary Artery Dissection Managed Conservatively

**DOI:** 10.1155/cric/7144164

**Published:** 2024-12-19

**Authors:** Garen S. Kroshian, Matthew J. Cozzolino, Edeliz Flores, Sheilah A. Bernard

**Affiliations:** ^1^Department of Internal Medicine, Boston Medical Center, Boston, Massachusetts, USA; ^2^Department of Cardiovascular Medicine, Boston Medical Center, Boston, Massachusetts, USA

**Keywords:** acute coronary syndrome, chest pain, coronary angiography, dissection

## Abstract

The management of spontaneous coronary artery dissection (SCAD) presents challenges and uncertainties. We present a case of a 54-year-old woman who developed SCAD in the three primary coronary artery territories including the distal left anterior descending artery (LAD), a diagonal branch, the first and second obtuse marginals (OMs), and the midright coronary artery (RCA). She was managed conservatively without procedural intervention, and follow-up coronary angiography demonstrated complete recovery.

## 1. Introduction

The management of spontaneous coronary artery dissection (SCAD) is an evolving area. There is a lack of randomized control trials that study the difference in outcomes between conservative treatment and intervention. Furthermore, the management of SCAD involving multiple coronary arteries is not well-established. We present a patient with multivessel SCAD with at least three different types of dissection who was treated successfully with conservative management.

## 2. History of Presentation

A 54-year-old woman presented to the emergency department with recurrent episodes of sudden onset sharp chest pain lasting minutes over the course of 1 day after receiving distressing news of the death of a family member, culminating in severe persistent pain lasting hours.

**Figure 1:**
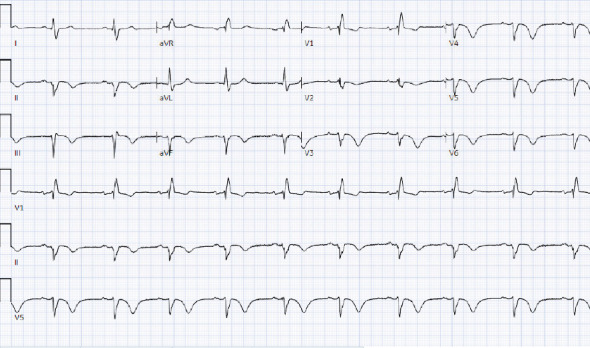
ECG acquired approximately 26 h after presentation showing new T wave inversions.

On arrival, blood pressure was 180/100 mmHg; otherwise, all vital signs were within normal limits. Physical exam was unremarkable. The patient did not have features consistent with connective tissue disease such as joint laxity, arachnodactyly, skin hyperextensibility, or marfanoid habitus. The initial electrocardiogram (ECG) at presentation revealed chronic right bundle branch block and left anterior fascicular block as well as new T wave inversions in the anterior leads and inferior leads. Serial ECGs showed progression of T wave inversions (Figure 1). Initial high sensitivity troponin was 207 ng/L (positive > 14 in women) and later reached a peak of 1280 ng/L.

## 3. Past Medical History

The patient had a history of obesity and underwent sleeve gastrectomy 2 weeks prior to presentation. She also had a history of heart failure with preserved ejection fraction, type two diabetes, hypertension, stage three chronic kidney disease, obstructive sleep apnea, and uterine fibroids with abnormal uterine bleeding. Notably, 2 years prior to presentation, she had a pharmacologic nuclear stress test suggestive of a small area of ischemia in the apical septum and was medically managed for coronary artery disease. She had never previously undergone coronary angiography.

## 4. Differential Diagnosis

The primary differential diagnosis for chest pain with evidence of acute myocardial ischemia is acute coronary syndrome, characterized by atherosclerotic plaque rupture with thrombus formation. An anatomically based differential can include other less common causes of coronary artery occlusion such as aortic and coronary artery dissections, coronary vasospasm, coronary artery embolism, and microvascular dysfunction.

## 5. Investigations

**Figure 2:**
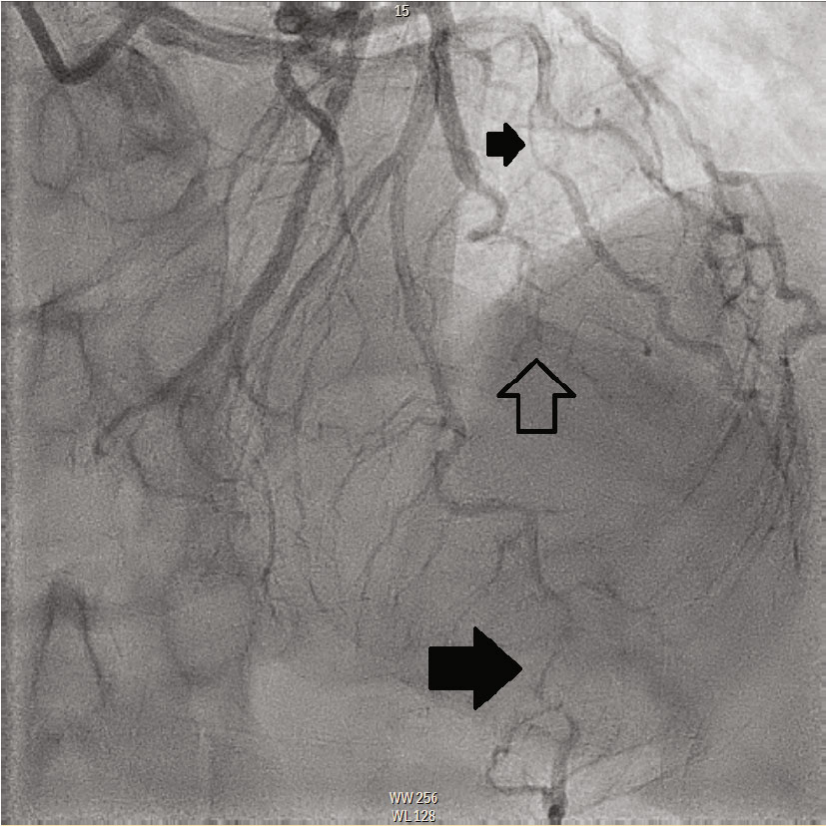
Anterior–posterior cranial view demonstrating a long diffuse lesion of the distal LAD (large black arrow), long tapering lesion leading to complete occlusion of an OM branch (hollow arrow), and short lesion of a diagonal branch (small black arrow).

**Figure 3:**
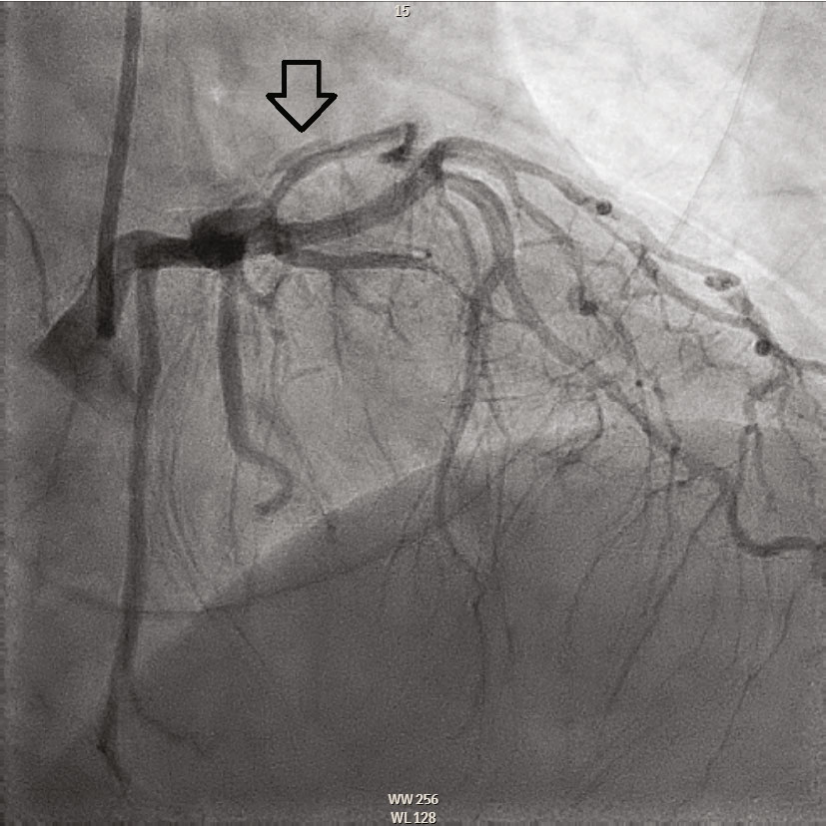
Right anterior oblique cranial view demonstrating a dissection plane with true and false lumen in an OM branch (hollow arrow).

**Figure 4:**
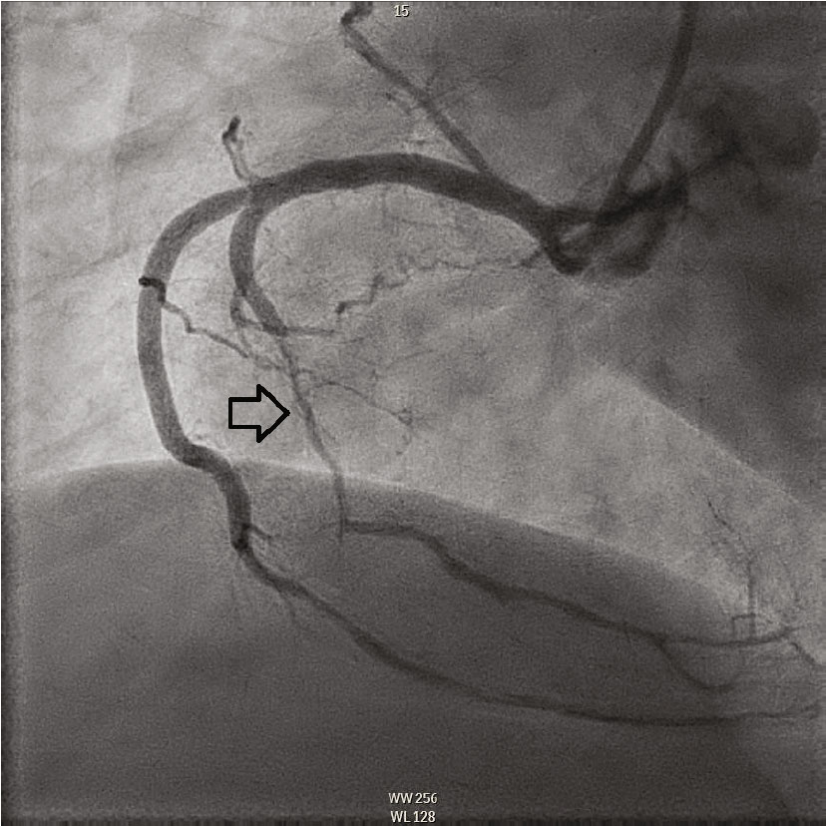
Left anterior oblique view demonstrating a long diffuse lesion in mid-RCA (hollow arrow).

The patient underwent coronary angiography on Hospital Day 1. She was found to have spontaneous dissections involving the distal left anterior descending artery (LAD), a diagonal branch, the first and second obtuse marginals (OMs), and the midright coronary artery (RCA) (Figures 2, 3, and 4; Supporting Information (available here)). More specifically, she had 100% distal LAD lesion, 90% first diagonal lesion, 50% 1^st^ OM lesion, 100% second OM lesion, and 70% mid-RCA lesion. Echocardiogram showed normal ventricular function without wall motion abnormalities.

Coronary computed tomography angiography (CTA) was attempted for future disease monitoring but was technically limited. For evaluation of other vasculopathies such as fibromuscular dysplasia, CTA of the head, neck, chest, abdomen, and pelvis was done that showed tortuosity of her vertebral arteries but no significant stenoses.

## 6. Management

The patient's chest pain resolved without intervention prior to undergoing angiography. She had received intravenous heparin prior to angiography, and this was discontinued thereafter. Revascularization options were deferred given complete resolution of chest pain, hemodynamic stability, and normal left ventricular function on transthoracic echocardiogram (completed on Hospital Day 2). She was transitioned from metoprolol to carvedilol to aid with blood pressure control. Her other home cardiac medications lisinopril, spironolactone, atorvastatin, and aspirin were continued.

On Hospital Day 3, the patient had a 45-s episode of ventricular tachycardia without symptoms. She had no further episodes of sustained ventricular tachyarrhythmias or recurrence of chest pain during the hospitalization. She was discharged home on Hospital Day 5.

**Figure 5:**
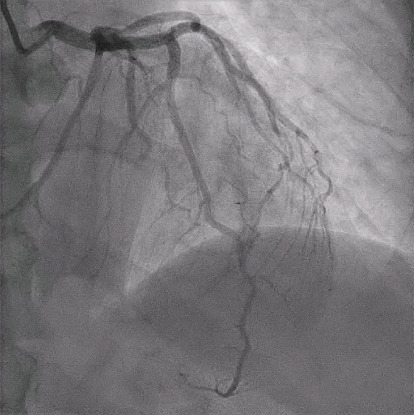
Anterior–posterior cranial view from follow-up coronary angiography demonstrating a normal-caliber left main coronary artery and branches.

**Figure 6:**
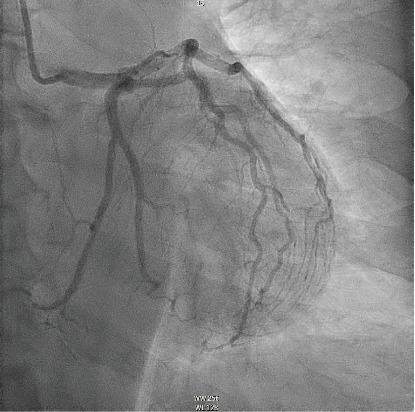
Right anterior oblique caudal view from follow-up coronary angiography demonstrating a normal-caliber left main coronary artery and branches.

**Figure 7:**
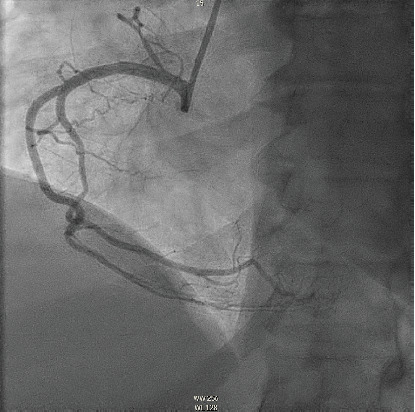
Left anterior oblique view from follow-up coronary angiography demonstrating normal-caliber RCA and branches.

She has had no further hospitalizations, recurrent SCAD, clinically evident arrhythmia, or worsening heart failure over the 6 months since her index presentation. For preoperative evaluation for uterine fibroid intervention, she underwent repeat coronary angiography 10 weeks after her discharge that showed complete healing of her coronary vasculature (Figures 5, 6, and 7; Supporting Information).

## 7. Discussion

As in our patient, SCAD is often associated with emotional stress and most commonly occurs in women less than 50 years [1, 2]. Testing for fibromuscular dysplasia is an important consideration in patients with SCAD, as it is a common disease association. Other considerations should include connective tissue disorders such as Marfan or Ehlers–Danlos syndromes. Our patient had no evidence of fibromuscular dysplasia on CT imaging and had no clinical features to suggest connective tissue disorder. Pregnancy, hormone therapy, and oral contraceptives have also been associated with SCAD, though not with our patient's presentation.

Only 9%–23% of SCAD cases involve multiple vessels. It is unclear whether non-ST elevation myocardial infarction, as our patient experienced, is the most common presentation of SCAD and whether the presence or absence of ST elevation should affect management [3, 4]. Severe ventricular arrhythmias are relatively rare with SCAD, with one study showing an occurrence of 3.9% in the 30 days following presentation [5].

SCAD can be classified into different types based on angiographic findings. Type 1 SCAD exhibits contrast staining of the arterial wall with multiple radiolucent lumens. Types 2 and 3 include the presence of intramural hematoma, Type 2 being diffuse long and smooth stenoses and Type 3 being tubular or focal stenosis that may mimic atherosclerosis. Some literature describes a fourth type of SCAD as defined as total occlusion of a vessel that does not meet the criteria for the other types of dissection [6]. Our patient demonstrated at least three different types of SCAD, including Type 1 in the first OM branch, Type 2 in the LAD and RCA, Type 3 in the diagonal branch, and Type 4 in the second OM branch (Figures 2, 3, and 4).

Recurrence rates of SCAD vary. Some evidence raises concern for early recurrence of dissection within the first 6 days of presentation [7]. A recent systematic review that assessed 13 different cohorts with follow-up times ranging from 7 to 75 months found the recurrence rate of SCAD to be as high as 31% [8]. However, in-hospital mortality for SCAD is low. Observational data have shown angiographic “healing” in 70%–97% of patients who underwent repeat coronary angiography weeks to months after index presentation [9, 10].

A primary question in the acute management of SCAD is whether patients should undergo revascularization, which may be with percutaneous coronary intervention (PCI) or surgical coronary artery bypass grafting (CABG). To date, there are no randomized controlled studies to guide these decisions. There are hypothesized concerns related to the pathophysiology of SCAD affecting revascularization. The underlying vasculopathy that predisposes to the development of SCAD may also predispose to PCI-related complications. SCAD lesions tend to be long and difficult to stent, and efforts to do so risk propagating the dissection further. Conversely, data on CABG following SCAD shows high rates of both venous and arterial graft failure. This is hypothesized to be due to competitive flow in the native coronary arteries once the dissected arteries have healed [9]. Though two large meta-analyses did not reveal differences in clinically relevant outcomes in patients undergoing revascularization versus conservative management, these studies did not stratify patients by severity of illness [10, 11]. Patients with more severe presentations were more perhaps more likely to undergo revascularization leading to confounding of outcome results. In contemporary practice, initial conservative management is generally preferred over revascularization in the absence of high-risk features such as hemodynamic or electrical instability, left main coronary artery involvement, or ongoing ischemia [12, 13]. Recent guidelines note that revascularization may also be considered for SCAD patients who fail conservative management, but routine revascularization in SCAD patients is considered harmful [14].

In our patient's case, the decision was made for conservative management given the tendency for SCAD lesions to self-resolve over time as well as the significant risk of revascularization attempts, whether percutaneous or surgical. Additionally, she lacked high-risk features at the time of angiography; she had no ongoing chest pain, unstable ventricular arrhythmia, heart failure or cardiogenic shock, or left main coronary artery involvement. Patients with high-risk features may prompt the operator to pursue emergent revascularization, and decisions should be made on an individualized basis.

There is limited data to guide the long-term medical management of patients with SCAD. The role of dual antiplatelet therapy (DAPT) is unclear though perhaps evolving. The 2018 American Heart Association Scientific Statement on SCAD indicates there is some consensus that patients should receive at least 1 year of aspirin therapy. However, various expert recommendations on DAPT range from no use to short-term use (1–3 months) to longer term use (at least 1 year) [3]. More recent registry data, though, has indicated that DAPT may be associated with higher rates of adverse cardiovascular events [15]. Further investigations are needed to define the role of antiplatelet therapy more clearly in SCAD. Beta-blockers, meanwhile, have been shown in a recent retrospective cohort study to decrease the risk of SCAD recurrence [1]. Other therapies such as statins, angiotensin-converting enzyme inhibitors, and nitrates do not have defined roles in management. Finally, SCAD-specific cardiac rehabilitation programs have shown benefit [16].

Patients with prior SCAD who later require cardiac testing for evaluation of new symptoms or preoperative clearance, like our patient, also present an area of challenges. Coronary angiography in these patients carries a risk of iatrogenic coronary artery dissection that is well documented [17, 18]. Given this circumstance, there has been interest in noninvasive imaging, particularly CTA, in the management of SCAD. Spatial resolution, however, has been cited as a limiting factor for CTA use in SCAD diagnosis; visualization of a dissection flap or intramural hematoma appears more technically limited than visualizing typical atherosclerotic lesions. While there have been reports of CTA documenting the healing of SCAD lesions, its overall accuracy in assessing SCAD healing remains uncertain [19]. Further experience with CTA as technological innovation continues may better define its role in the follow-up of SCAD patients.

## 8. Conclusions

Our case demonstrates that extensive multivessel SCAD may be managed conservatively without PCI or CABG, adding to the evidence of the natural history of spontaneous healing seen with SCAD.

## Data Availability

The authors have nothing to report.

## Nomenclature

CABG: coronary artery bypass grafting
CTA: computed tomography angiography
DAPT: dual antiplatelet therapy
ECG: electrocardiogram
LAD: left anterior descending artery
OM: obtuse marginal
PCI: percutaneous intervention
RCA: right coronary artery
SCAD: spontaneous coronary artery dissection

## Supporting Information

Supporting Information Additional supporting information can be found online in the Supporting Information section. Left heart catheterization films showing different views of our patient's spontaneous coronary artery dissections. The LCA RAO cranial view shows a dissection plane with true and false lumen in an OM branch. The LCA straight CRA shows a long diffuse lesion of the distal LAD and long tapering lesion leading to complete occlusion of an OM branch. This view also shows a short lesion of a diagonal branch. The RCA LAO straight view demonstrates a long diffuse lesion in the mid-RCA. Ten weeks after discharge, the patient had a repeat coronary angiogram that showed resolution of coronary dissections. The repeat coronary angiogram films are labeled RCA small LAO CRA, LCA RAO CAU, and LCA small RAO CRA.
