# Supplementary material for: Triple-Vessel Spontaneous Coronary Artery Dissection Managed Conservatively
Source: Case Rep Cardiol. 2024 Dec 19;2024:7144164. doi: 10.1155/cric/7144164 (PMC11671644; doi:10.1155/cric/7144164)
Supplement: Supporting Information — Additional supporting information can be found online in the Supporting Information section. Left heart catheterization films showing different views of our patient's spontaneous coronary artery dissections. The LCA RAO cranial view shows a dissection plane with true and false lumen in an OM branch. The LCA straight CRA shows a long diffuse lesion of the distal LAD and long tapering lesion leading to complete occlusion of an OM branch. This view also shows a short lesion of a diagonal branch. The RCA LAO straight view demonstrates a long diffuse lesion in the mid-RCA. Ten weeks after discharge, the patient had a repeat coronary angiogram that showed resolution of coronary dissections. The repeat coronary angiogram films are labeled RCA small LAO CRA, LCA RAO CAU, and LCA small RAO CRA. [file 7144164.f1.docx]

**Supplementary Section**

<https://drive.google.com/drive/folders/1ihDlhSMlg2iXCcmMHRKu0Wtka-Neqqk8?usp=drive_link>

Left heart catheterization films showing different views of our patient’s spontaneous coronary artery dissections. The LCA RAO Cranial view is showing dissection plane with true and false lumen in an OM branch. The LCA Straight CRA is showing long diffuse lesion of the distal LAD and long tapering lesion leading to complete occlusion of an OM branch. This view also shows a short lesion of a diagonal branch. The RCA LAO Straight view is demonstrating a long diffuse lesion in the mid RCA.

Left heart catheterization films labeled RCA small LAO CRA, LCA RAO CAU, and LCA small RAO CRA are from interval coronary angiogram 10 weeks after discharge that show resolution of coronary dissections.
